# Supplementary material for: An atlas of neural crest lineages along the posterior developing zebrafish at single-cell resolution
Source: eLife. 2021 Feb 16;10:e60005. doi: 10.7554/eLife.60005 (PMC7886338; doi:10.7554/eLife.60005)
Supplement: Figure 1—source data 2. — References cited: Barske et al., 2016; Carney et al., 2006; Ding et al., 2013; Du et al., 2003; Dutton et al., 2001; Farnsworth et al., 2020; Feregrino et al., 2019; Gaudet et al., 2011; Heanue and Pachnis, 2008; Knight et al., 2003; Lister et al., 1999; Ludwig et al., 2004; Lu et al., 2019; Luo et al., 2001; Minchin and Hughes, 2008; Nakamura et al., 2016; Nord et al., 2016; Parichy et al., 2000; Petratou et al., 2018; Petratou et al., 2019; Quigley and Parichy, 2002; Saunders et al., 2019; Shepherd et al., 2004; Soldatov et al., 2019; Sperber and Dawid, 2008; Sperber et al., 2008; Stewart et al., 2006; Taylor et al., 2016; Thisse et al., 2001; Thisse and Thisse, 2004; Thisse and Thisse, 2005; Uyttebroek et al., 2010; Yelon et al., 2000. [file elife-60005-fig1-data2.pdf]

| Time Point | Cluster ID # | Major Cell Type Classifier | Subtype | Identifier genes | # of Cells in Cluster | Citations |
|---|---|---|---|---|---|---|
| 48-50HPF | cl0 | Neuronal | Autonomic Neuronal Progenitor | **phox2bb**, **tfap2a/b**, **elavl3/4**, **phox2a**, tuba2, ncam1a, draxin,sncb,fez1,cdh2 | 201 | Farnsworth et al., 2020; Soldatov et al., 2019 |
| 48-50HPF | cl1 | Mesenchyme | Mesenchymal Progenitor | **prrx1a**, **snai1a**, **twist1a**, **foxc1b**, mcm5, pcna, mcm7,cdh11, meox1 | 160 | Soldatov et al., 2019 |
| 48-50HPF | cl2 | Mesenchyme | Chondrogenic Mesenchyme Proliferative 1 | **dlx2a**, **prrx1a/b**, **twist1a/b**, **barx1**, cdk1,ccna2 | 154 | Soldatov et al., 2019; Sperber et al., 2008; Sperber and Dawid, 2008; Ding et al., 2013; Barske et al., 2016 |
| 48-50HPF | cl3 | Mesenchyme | Chondrogenic Mesenchyme Proliferative 2 | **barx1, dlx2a**, **mcm6**, **prrx1b**, chaf1a,pcna,hand2 | 129 | Soldatov et al., 2019; Sperber et al., 2008; Sperber and Dawid, 2008; Ding et al., 2013; Barske et al., 2016 |
| 48-50HPF | cl4 | Mesenchyme | Chondrogenic Mesenchyme Stem-like | **prrx1a**, **dlx2a**, **barx1**, **twist1a**, rpa1, fen1, rpa2, uhrf1, id3, chaf1a | 108 | Soldatov et al., 2019; Sperber et al., 2008; Sperber and Dawid, 2008; Ding et al., 2013; Barske et al., 2016 |
| 48-50HPF | cl5 | Neural Crest | Migratory Neural Crest/ Enteric Neural Crest | **sox10**, **foxd3**, **crestin,tfap2a,** mmp17b, pcdh10a,phox2bb, ret, ngfrb, gfra1a, | 93 | Dutton et al., 2001; Luo et al., 2001; Knight et al., 2003; Stewart et. al., 2006 |
| 48-50HPF | cl6 | Mesenchyme | Mesenchyme Differentiating 1 | **foxc1a**, **twist1a, tagln2,aldh1a2,** rdh10a, meis3, cyp26a1, hand2, tpm4a, actb1 | 88 | Soldatov et al., 2019 |
| 48-50HPF | cl7 | Neuronal | Central Nervous System neuron | **gad1b,gad2, slc6a5,slc32a1,gata2a,**irx1b, elavl3, elavl4, tuba2, phox2bb | 86 | Farnsworth et al. 2020 |
| 48-50HPF | cl8 | Pigment | Melanophore | **typr1a/b**, **mitfa**, **dct**, **pmela**, slc45a2, sox10, mlpha, tspan10, oca2 | 81 | Du et al., 2003; Lister et al., 1999; Ludwig et al., 2004; Quigley and Parichy, 2002 |
| 48-50HPF | cl9 | Mesenchyme | Chondrogenic Mesenchyme Migratory | **dlx2a, barx1**, **foxc1a**, **twist1a**, dlx4a, id2a, snai1a/b, twist3 | 77 | Soldatov et al., 2019; Sperber et al., 2008; Sperber and Dawid, 2008; Ding et al., 2013; Barske et al., 2016 |
| 48-50HPF | cl10 | Mesenchyme | Mesenchyme Differentiating 2 | **cdh11**, **foxc1a/b, prrx1a, meox1**, pdgfra, col2a1a, col9a1a, col9a2, sparc, colec12, reck | 74 | Soldatov et al., 2019 |
| 48-50HPF | cl11 | Mesenchyme | Mesenchymal Migratory | **cxcl12a, cxcl12b, rac1,reck**, foxc1a, twist1a, prrx1a, meox1, snai2 | 74 | Soldatov et al., 2019 |
| 48-50HPF | cl12 | Mesenchyme | Chondrogenic Mesenchyme Proliferative 3 | **prrx1b**, **twist1b**, **foxc1a**, **barx1**, cyp26a1, rdh10a, meis3, aldh1a2, mcm5, mcm6 | 61 | Soldatov et al., 2019 |
| 48-50HPF | cl13 | Neural | Neural Progenitor | **notch1a**, **dla/b**, **olig4**, **elavl3**, phox2ba, neurod4, scrt2, phox2bb, ncam1a | 61 | Farnsworth et al., 2020 |
| 48-50HPF | cl14 | Mesenchyme | Fin Bud | **tbx5a**, **hand2**, **hoxa13a/b, prrx1a/b,** foxc1b, hoxd13a, pcna | 58 | Yelon et al., 2000;Lu et al., 2019;Nakamura et al., 2016;Feregrino et al., 2019 |
| 48-50HPF | cl15 | Glial | Peripherial Glial Progenitor | **sox10**, **olig1/2**, **fabp7a**, **notch1a**, sox3, nkx2.2a, pou3f1,plp1a,gfap,s100b | 44 | Soldatov et al., 2019; Carney et al. 2006 |
| 48-50HPF | cl16 | Otic | Otic Epithelium | **sox10, cdh1, otomp**,**cldnb**, cldn7b, cldnh, epcam, aldh1a3,vamp8 | 23 | Thisse et al., 2004; Thisse et al., 2005 |
| 48-50HPF | cl17 | Neuronal | Sensory Neuronal Progenitor | **ngfrb**, **neurod1**, **neurod4**,**vim** neurog1, grfra1a, six1a/b, elavl4, isl2a/b, pou4f1 | 23 | Soldatov et al., 2019 |
| 48-50HPF | cl18 | Muscle | Muscle | **ckmb, actc1b, tnnc2, tnnt3a**, pvalb2, tnnt3b, ak1, tpma, desma | 13 | Thisse et al., 2004 |
| | | | | | | |
| 68-70HPF | cl0 | Mesenchyme | Chondrogenic Differentiating Mesenchyme 1 | **barx1, sparc, prrx1a/b**, **twist1a/b**, pdgfra, col1a2, col6a1, col5a1 | 281 | Soldatov et al., 2019; Sperber et al., 2008; Sperber and Dawid, 2008; Ding et al., 2013; Barske et al., 2016 |
| 68-70HPF | cl1 | Mesenchyme | Fin Bud | **tbx5a, hand2,prrx1a/b , hoxd13a,** pcna, foxc1a/b, meox1,hoxa13a/b | 254 | Yelon et al., 2000;Lu et al., 2019;Nakamura et al., 2016;Feregrino et al., 2019 |
| 68-70HPF | cl2 | Mesenchyme | Mesenchymal Migratory | **meox1, prrx1a**/b**, foxc1b**, snai1a, cdh11, rac1a, myl9b, **twist1a/b** | 229 | Soldatov et al., 2019 |
| 68-70HPF | cl3 | Neural | Neural Progenitor | **fabp7a, foxd3**, **sox10, phox2bb**, her4.2, mdka, her4.1, ccnd2a, ngfrb, tuba8l3 | 228 | Farnsworth et al. 2020 |
| 68-70HPF | cl4 | Pigment | Melanophore | **mitfa**, **pmela**, **tyrp1a**/b, **dct**, oca2, mlpha, tspan10, tfap2e | 213 | Du et al., 2003; Lister et al., 1999; Ludwig et al., 2004; Quigley and Parichy, 2002 |
| 68-70HPF | cl5 | Neuronal | Sympatho-enteric Progenitor | **ascl1a, phox2bb**, **phox2a**, **hand2**, insm1a/b, dpysl3, ret, ngfrb, gfra1a,elavl3/4 | 202 | Thisse et al., 2004;Heanue and Pachnis, 2008 |
| 68-70HPF | cl6 | Mesenchyme | Chondrogenic Differentiating Mesenchyme 2 | **barx1**, **prrx1a**, **foxc1a/b, twist1a**, col1a2, col5a1, cdh11, dlx5a, sparc,meox1 | 191 | Soldatov et al., 2019 |
| 68-70HPF | cl7 | Mesenchyme | Fin Bud | **tbx5a, hand2, hoxa13a/b,prrx1a/b** , hoxd13a, pcna, sox10 | 178 | Yelon et al., 2000;Lu et al., 2019;Nakamura et al., 2016;Feregrino et al., 2019 |
| 68-70HPF | cl8 | Mesenchyme | Chondrogenic Proliferative Mesenchyme | **prrx1a/b**, **foxc1a**, **twist1a/b**, **barx1**,col2a1a, pcna, cdc6, ccnb2 | 98 | Soldatov et al., 2019 |
| 68-70HPF | cl9 | Muscle | Pigmented Muscle | **tnnt3b, mitfa**, **tyrp1a**,**tpma**, ckmb, gch2, dct, tnnc2, myl1, | 93 | Thisse et al., 2004; Thisse et al., 2005 |
| 68-70HPF | cl10 | Neural | Neural Progenitor | **sox10**, **foxd3, ascl1a, aurkb**, ncam1b, ccna2, tpx2, phox2bb, ret, elavl3/4 | 76 | Farnsworth et al. 2020 |
| 68-70HPF | cl11 | Otic | Otic Epithelium | **cldne**, **vamp8, cldnb**, **epcam**, cldn7b, cldnh, cdh1 | 70 | Thisse et al., 2004, Thisse et al., 2001 |
| 68-70HPF | cl12 | Neuronal | Enteric Neuron | **elavl3/4, phox2bb, gfra1a, ret,** fgf13b, vip, vipb, nos1, ngfrb, hoxb5b | 44 | Shepherd et al., 2004; Heanue and Pachnis, 2008; Uyttebroek et al., 2010; Taylor et al., 2016; Gaudet et al., 2011 |
| 68-70HPF | cl13 | Pigment | Pigment Progenitor | **sox10**, **tfec**, **tfap2e, mitfa,** zeb2a, cdh1, mlpha, pax3a, trpm1b, gpx3 | 43 | Saunders et al., 2019 |
| 68-70HPF | cl14 | Glial | Schwann Cells | **sox10**, **mbpa/b, pou3f1, egr2b,** fabp7a, erbb3a, plp1a, cx27.5, gldn, mag | 42 | Farnsworth et al. 2020 |
| 68-70HPF | cl15 | Pigment | Xanthophore | **xdh**, **pax7a/b**, **gch2, aox5** | 39 | Nord et al., 2016; Parichy et al., 2000; Saunders et al., 2019; Minchin and Hughes, 2008; Lister et al., 1999 |
| 68-70HPF | cl16 | Pigment | Iridiophore | **atic, gpnmb, tfec, pnp4a** | 32 | Higdon et al., 2013; Lister et al., 2011; Petratou et al., 2018, 2019 |
| 68-70HPF | cl17 | Mesenchyme | Differentiating Mesenchyme 1 | **twist1a**,**prrx1a,colec12**, **sparc**, mmp2, msx2b, col5a1, col1a1a, pdgfra, tnc | 25 | Soldatov et al., 2019 |
| 68-70HPF | cl18 | Pigment | Proliferative Melanophores | **mitfa, pmela, tyrp1a/b, aurkb,** pcna, cenph, mlpha, dct, gpr143, tfap2e | 19 | Du et al., 2003; Lister et al., 1999; Ludwig et al., 2004; Quigley and Parichy, 2002 |
| 68-70HPF | cl19 | Muscle | Muscle | **ckmb, tpma, tnnc2, tnnt3b**, actc1b, cavin4a, myom1a, neb, flnca, desma, | 18 | Thisse et al., 2004 |
| 68-70HPF | cl20 | Mesenchyme | Chondrogenic Mesenchyme Migratory | **barx1, twist1a**, **foxc1a/b, snai1a**, dlx3b, mmp2, sparc, snai2, dlx2a, sox9a | 16 | Soldatov et al., 2019 |
| 68-70HPF | cl21 | Unidentified/Spurious | Unidentified/Spurious | anxa2b, cldnc, faah2b, vil1, fabp1b.1, plac8.1 | 13 | |
| 68-70HPF | cl22 | Mesenchyme | Differentiating Mesenchyme 2 | **rac2**, **sparc**, **col18a1**, **col2a1b,** col1a1a, lama5, thy1 | 6 | Soldatov et al., 2019 |
